# Virtual reality: a promising instrument to promote sail education

**DOI:** 10.3389/fpsyg.2023.1185415

**Published:** 2023-07-26

**Authors:** Fa Ji, Xingjian Zhang, Shan Zhao, Qun Fang

**Affiliations:** ^1^School of Physical Education, Qingdao University, Qingdao, China; ^2^Development Center for Water Sports, Qingdao University, Qingdao, China

**Keywords:** virtual reality, sailing, skill transfer, physical education, pedagogy

## Abstract

Sailing has gained an increasing attention among children and adolescents in China, which raised a strong need for sail courses through physical education (PE). However, challenges in teaching practice arise with rapid development of the sport. In the current study, we proposed a perspective that virtual reality (VR) technology makes high-quality sail education accessible for students. Critical analysis summarized the prominent features that enhance sail education, including immersive experience, interactive learning, the first-person view, and practice under well-controlled conditions. Further, research on VR sport training indicated successful transfer from virtual environment to real situation. Specifically, significant improvement in skill performance and tactical behaviors were identified, which was attributed to the enhanced perception-action coupling after VR training. Additionally, VR-based coding programs provide students with affordances of designing the virtual environment. The content design education promotes comprehension and application of knowledge and theories when students develop the simulated environment with a high level of presence. Therefore, VR technology is a promising instrument to meet the increasing demand on sail education. While VR enriches educational resources for a large class size, the interdisciplinary feature of VR-based sail course can attract students with different study interests and backgrounds to the class.

## 1. Introduction: opportunities and challenges in sail education

Sailing has become a popular sport in China since 2008 Beijing Olympic Games. In coastal cities such as Qingdao, sailing has been embedded in the school curriculum. Educational resources nowadays are available for students to learn knowledge and skills via sail classes at school. In the meantime, sailing clubs provide children and adolescents with a variety of off-school services from sail experience for recreation to regular training for competition.

The increasing popularity of sailing can be attributed to awareness of the benefits in physical and psychological development. As a primary form of outdoor and adventure education, sailing is more of a powerful educational approach than of adventurous recreation (Mcculloch et al., 2010). Qualitative research by means of focus group summarized benefits of sailing for children in self-confidence and competence, key personal and interpersonal skills, specific life skills, academic performance, physical fitness, and mental health (Cotterill and Brown, 2018). Additionally, a pre-post study involving 147 adolescents was conducted to examine influence of sail education programs on adolescents. Significant improvement was found in self-concept of competence and social skills after the sail experience (Capurso and Borsci, 2013). Further evidence has shown that water-based environment is favorable to natural settings without water (Depledge et al., 2011). A meta-analysis identified positive effects of green environment (e.g., urban green, countryside/farmland, forest and woodland, waterside, and wild habitats) on self-esteem and mood, but the presence of water generated the largest effect size (Barton and Pretty, 2010).

Physical education (PE) is an important approach to promoting sailing among the young generations in China. However, challenges also arise in teaching practice during rapid development of the sport. The increased class size raises a major challenge on availability of resources for students to gain adequate sailing experience. Opportunities of operation and practice can be reduced in a larger class size, which demands supplementary teaching strategies to ensure quality of the course. In addition, traditional sail course begins with teaching knowledge out of water, and then progresses to application and practice (Morales-Belando and Arias-Estero, 2017). The course integrates mathematics, physics, geometry, and other disciplines, which increases difficulty in learning. Weeks of lecture-based study can be mentally exhausting and boring for children and adolescents, thus reducing teaching and learning effects. Another main challenge lies in transfer of knowledge and skills to practice. It is common to see that high uncertainties of sailing on the sea make novices struggling, including those who showed outstanding performance in theoretical learning. For the considerations of the above-mentioned challenges, innovative strategies are needed to enrich educational affordances, enhance motivation of learning, and facilitate transition from theory to practice.

Virtual reality (VR) technology using head-mounted display (HMD) is portable, convenient, and affordable (Schiza et al., 2019), which has been widely applied to medical and language education (Chen et al., 2020; Parmaxi, 2023). A simulated environment providing learners with authentic learning experience can enhance both motivations and engagement in task performance, thus offering potential solutions to the challenges in teaching practice (Rey et al., 2022). The current study aims to investigate feasibility and efficacy of applying VR technology to sail education. Critical analysis focused on the prominent features of VR which facilitate acquisition of sailing knowledge and skills. In addition, efficacy of VR in sail education was evaluated by evidence regarding transfer of learning from virtual environment to real task. Further discussions provided insights into VR course design, leading to the perspective that integration of VR into sail education is a promising approach to diverse PE content and effective pedagogical strategies.

## 2. Features of VR technology to facilitate sail education

VR technology generates immersive experience with a high level of representation to the real world. The extent of immersion is measured by presence which refers to subjective feeling of “being there” (Sanchez-Vives and Slater, 2005). Simulating the real experience is important to sail education given that perception of wind speed, wind direction, current, and wave can hardly be developed by knowledge learning in a classroom. VR presents theoretical knowledge in the form of 3D practical scenarios (Plotzky et al., 2021). Sailing simulators based on mathematic models have accounted for a variety of variables (Mulder and Verlinden, 2013; Angelou and Spyrou, 2021) which enable novices to gain valuable hands-on experience in VR at the early stage of learning. Research has shown that virtual environment with the higher immersive quality induces the greater psychological presence and similar responses to the real situations (Bowman and Mcmahan, 2007; Miles et al., 2012). The immersive experience can familiarize novices with the environment and lower the impact of nervousness and anxiety on performance. Indeed, mental readiness is essential for novices due to the high pressure and tight temporal constraints in sailing. Researchers used VR technology to prepare athletes for high-pressure situations such as soccer penalty kick (Stinson and Bowman, 2014). The significant reduction in sport-induced anxiety implies promising applications of VR to facilitate adaptation to the real sailing.

In addition to the immersive experience which helps learners to be prepared with the real task, another remarkable feature of VR is to provide interactive experience between the learners and the virtual environment (Neumann et al., 2018). In a scoping review regarding VR training on team ball sports performance, researchers summarized three types of interactions, including interaction with ball, interaction with other players, and interaction with both ball and players, which showed positive effects on both perception and performance (Faure et al., 2020). In the research on rowing simulation, interactions between oar and water were essential factors which induced comparable skill gains to the real training (Rauter et al., 2013). Accordingly, it is reasonable to expect positive effects of VR sailing should interactions between actions and corresponding outcomes be represented in the sailing simulation.

The first-person view in HMD enhances immersion and interactive experience, which plays an essential role in training effects and sport performance (Covaci et al., 2015). Petit and Ripoll (2008) found that experienced soccer players made faster and more accurate decisions in the first-person view than in the broadcast point of view. In the case of sail education, the first-person view makes the learning process more active given the fact that learners can freely observe surroundings and collect perceptual information from the virtual environment. In this sense, learning in VR becomes an exploratory process. Taking course keeping in the upwind sailing as an example, theories only give learners the optimal angle to sail in a desired direction. It is also important for learners to gain insights into various outcomes of adjusting the sail at different angles. The exploratory learning by interacting with virtual environment can be greatly helpful for novices to enrich sailing experience from the simulated operation. Therefore, VR technology enables the principle of learning by doing to be implemented in sail education, which is a prominent advantage over traditional approaches.

The high level of control over the designed task and environment is of great value in VR-based training (Bideau et al., 2010; Zaal and Bootsma, 2011). It is suitable to use VR as a supplementary approach to the on-site training which is expensive, dangerous, or difficult to replicate in real life (Oagaz et al., 2022). For sail education, standardized procedures can be simulated to provide affordances for repetitions on specific skills and performance (Mulder and Verlinden, 2013). VR training is not only for the realistic situations, but also for extreme, unrealistic conditions to “over-prepare” trainees for the real situation (Oagaz et al., 2022). In addition to the environmental design, the customized sail simulator setup also allows a full control over feedback in both quantity and quality to guide learners during training (Faure et al., 2020). For example, a checklist for safety check before departure can be transferred to visual and verbal instructions. The instructions can be provided in concurrent with each step to assist students in completing the task. Therefore, the above-mentioned characteristics of VR technology, including immersive experience, interactive learning, the first-person view, and affordances for adequate repetitions under well-controlled conditions, substantiate the feasibility of integrating VR into sail education.

## 3. Efficacy of skill development: transfer from virtual to real environment

Transfer of learning from virtual environment to real task is a determinant for the efficacy of VR-based practice. A wide application of VR to sail education should be justified by empirical evidence for successful transfer. Harris et al. (2021) examined validity of a VR golf putting simulator. Participants reported a high degree of presence in the virtual environment, which validated using VR technology to simulate real golf putting. Additionally, in a study which compared movement patterns in VR with those in real situations, similar stance patterns were identified when tennis players performed groundstrokes, suggesting adequate representativeness of a virtual environment to the actual tennis performance (Le Noury et al., 2021). Cumulative evidence suggests a greater role of functional fidelity (e.g., tracking level, stereoscopy, and field of view) than physical fidelity (e.g., image and sound quality) in contributing to a high-quality virtual environment (Cummings and Bailenson, 2016). To increase functional fidelity, it is important to preserve the perception-action loop that is normally experienced in a real environment (Craig, 2013).

The extent of learning transfer to real situation is a critical consideration regarding the effectiveness of sport training via VR technology. Research has shown that VR facilitates acquisition of table tennis skills measured by quality of returned balls (Oagaz et al., 2022). In this study, novices were randomly assigned to either VR group or conventional training group. After five table tennis sessions, the VR group outperformed the control group in terms of speed and height of the returned ball, indicating improved returning technique. The findings led to the conclusion that VR is effective in improving complex sport skills and the learned skills can be transferred to enhance actual performance.

It is also an interest of study in the influence of VR on cognitive perception which is critical for sport performance. A recent study examined effects of using a VR system to assist basketball players in understanding and executing offensive tactics (Tsai et al., 2022). Participants learned and practiced four tactics which were provided by VR system, video clips, and instructions on tactic board. VR training induced greater improvement in accuracy of running path in the field tests than the other groups. The advantage of VR in tactic training is more evident when tactics involve with multiple steps and more players. For simple tactics, the conventional training approach by tactic board could provide adequate information. As tactical complexity increases, the immersive experience facilitates strategic imagery and comprehension of tactics which help the players with execution in the real situations.

A possible explanation to the positive transfer of VR training to actual performance is the enhanced perception-action coupling. In a baseball batting study, high school baseball players were allocated to one of the groups which received adaptive VR practice, VR practice, and real batting practice in addition to the regular baseball training (Gray, 2017). Adaptive VR practice was implemented by manipulating pitch parameters (e.g., spin, speed, pitch crossing height, and pitcher’s handedness) to match performance in each trial. Favorable batting performance was identified in the adaptive VR over the other groups, suggesting the positive effect of pitch recognition on actual performance. Therefore, evidence for the transfer of learning has been found in both behavioral (Oagaz et al., 2022) and cognitive performance (Tsai et al., 2022), which further implies an essential role of the enhanced perception-action coupling in the successful transfer from virtual environment to real situation (Gray, 2017).

Therefore, the main findings from the existing research substantiated the following statements: (1) VR technology is valid in simulating the real situation; (2) training in the simulated environment benefited actual performance in skill and tactical behaviors; and (3) enhanced perception-action coupling may contribute to the positive transfer from practice in the virtual environment to performance in the real task.

## 4. VR sailing design: a novel approach to enhance understandings

While sailing skills can be practiced in a simulated environment, understandings of knowledge can be enhanced via VR-based design education. This innovative approach allows teachers and students to collaboratively create course content, which stimulates new ways of thinking during the design activity (Denner et al., 2012). Such an idea was inspired by a VR-based coding education program for middle and high school students in South Korea (Shim and Lee, 2022). The researchers implemented a six-week coding course with an emphasis on energy education. By asking the students to design a VR theme park using future energy, the teachers helped the students to raise awareness of energy issues in the communities and improve problem solving abilities in energy saving. Significant benefits were reported in the students’ competence and educational satisfaction, thus providing evidence for the VR-based design education in promoting effectiveness of both teaching and learning.

Adequate simulation of sailing demands comprehensive understandings of the sport. Considerations regarding stereoscopy and principles of physics should be counted when designing virtual environment of sailing on the sea. The sail course may give particular attention to VR content design by encouraging students to develop code and algorithm. More importantly, interactions between the environmental factors (e.g., wind, wave, current) and outcomes (e.g., vessel direction and velocity) are essential for a high level of presence. Students can easily identify potential flaws in the coding if the outcome is not in line with expectation. Further corrections then should be conducted to make the virtual environment close to the real one.

**Figure 1:**
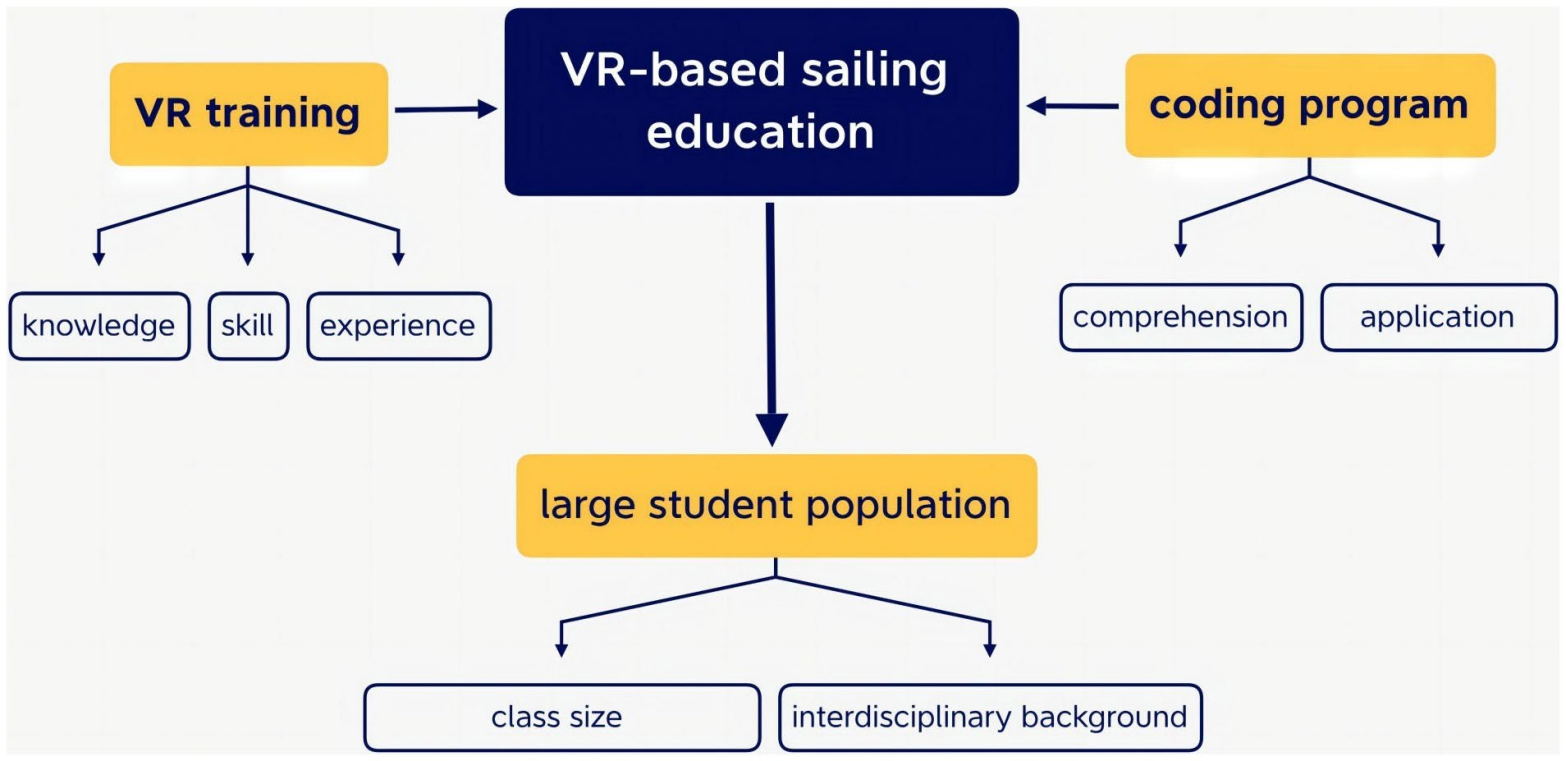
Framework of VR-based sail course.

In summary, a framework of VR-based sail course was developed in correspondence to the increasing need among the students in China (Figure 1). Sail education can be provided through VR training and coding program. While VR training facilitates the learning process in which knowledge, skills, and experience of sailing can be improved in the simulated environment, coding program enhances comprehension and application of sailing knowledge by designing the simulated environment. Therefore, the course framework allows sail education to accommodate a large student population. On the one hand, VR technology enriches educational affordances for a larger class size. On the other hand, the interdisciplinary feature allows students with a wide variety of study interests and backgrounds to take the course.

## 5. Conclusion

The increasing popularity of sailing in China results in the rapidly growing need for sail education at school. However, practical challenges also arise in considerations of the increasing class size, study motivation, and transition from theory to practice, which highlight the necessity of innovative pedagogical strategies to enhance sail education. VR is characterized by immersive experience, interactive learning, the first-person view, and affordances for adequate repetitions under well-controlled conditions. Additionally, empirical evidence from the existing research indicates successful transfer of learning from virtual environment to real task, which implies the efficacy of using VR for knowledge learning and skill development in sail education. VR technology not only provides opportunities for practice, but also offers a platform for VR content design. Coding programs help students to enhance comprehension and application while designing a simulated sailing environment. Therefore, VR is a promising instrument to meet the increasing demand on sail education by providing adequate educational resources for a large class size and making the class accessible for students with different study interests and backgrounds.

## Data availability statement

The original contributions presented in the study are included in the article/supplementary material, further inquiries can be directed to the corresponding author.

## Author contributions

FJ and XZ prepared the draft. FJ, SZ, and QF worked on revision and approved the submitted version. FJ, XZ, SZ, and QF collaborated in preparing the manuscript. All authors contributed to the article and approved the submitted version.

## Conflict of interest

The authors declare that the research was conducted in the absence of any commercial or financial relationships that could be construed as a potential conflict of interest.

## Publisher’s note

All claims expressed in this article are solely those of the authors and do not necessarily represent those of their affiliated organizations, or those of the publisher, the editors and the reviewers. Any product that may be evaluated in this article, or claim that may be made by its manufacturer, is not guaranteed or endorsed by the publisher.
